# The clinical value of circadian biofeedback in chronic heart failure

**DOI:** 10.1093/ehjdh/ztag036

**Published:** 2026-02-25

**Authors:** Valerie A A van Es, Ignace L J De Lathauwer, Freek J C van Blerck, Mayke M C J van Leunen, Danny A J P van de Sande, Rutger W M Brouwers, Mathias Funk, Emiliano Ricciardi, Giacomo Handjaras, Monica Betta, Hareld M C Kemps

**Affiliations:** Department of Cardiology, Máxima Medical Centre, Veldhoven/Eindhoven, the Netherlands; Department of Industrial Design, Eindhoven University of Technology, Eindhoven, the Netherlands; MoMiLab Research Unit, IMT School for Advanced Studies, Lucca, Italy; Department of Cardiology, Máxima Medical Centre, Veldhoven/Eindhoven, the Netherlands; Department of Industrial Design, Eindhoven University of Technology, Eindhoven, the Netherlands; Department of Cardiology, Máxima Medical Centre, Veldhoven/Eindhoven, the Netherlands; Department of Industrial Design, Eindhoven University of Technology, Eindhoven, the Netherlands; Department of Cardiology, Máxima Medical Centre, Veldhoven/Eindhoven, the Netherlands; Department of Industrial Design, Eindhoven University of Technology, Eindhoven, the Netherlands; Department of Cardiology, Máxima Medical Centre, Veldhoven/Eindhoven, the Netherlands; Department of Industrial Design, Eindhoven University of Technology, Eindhoven, the Netherlands; Department of Industrial Design, Eindhoven University of Technology, Eindhoven, the Netherlands; MoMiLab Research Unit, IMT School for Advanced Studies, Lucca, Italy; MoMiLab Research Unit, IMT School for Advanced Studies, Lucca, Italy; MoMiLab Research Unit, IMT School for Advanced Studies, Lucca, Italy; Department of Cardiology, Máxima Medical Centre, Veldhoven/Eindhoven, the Netherlands; Department of Industrial Design, Eindhoven University of Technology, Eindhoven, the Netherlands

**Keywords:** Heart failure, Circadian rhythms, ECG-actigraphy, Autonomic regulation, Wearables, Digital health

## Abstract

**Aims:**

Chronic heart failure (CHF) is characterized by impaired autonomic regulation and disrupted sleep–wake cycles, limiting recovery and daily function. CircAlign-HF, a wearable-guided circadian biofeedback tool providing personalized timing recommendations on activity, naps, and sleep, was developed. This study evaluated its physiological and clinical value in CHF.

**Methods and results:**

Twenty-one patients with stable CHF (median age 68 years, 90.5% male, NYHA II–III) completed a 3-week crossover protocol. Week 1 served as baseline, in Week 2 participants received general advice (30 min daily walk, 20 min nap, sleep hygiene), and in Week 3, participants were guided to align their walk, nap, sleep, and wake times with their circadian rhythm. Autonomic regulation was assessed using HRV over 24 h, and within 15 min windows centred on each participant’s peak daytime activity (diurnal acrophase) and deepest nocturnal rest (nocturnal nadir), capturing activity-related and sleep-related autonomic function. Patient-centred outcomes and adherence were evaluated using a structured questionnaire.

General advice improved 24 h autonomic regulation: the root mean square of successive differences (rMSSD) increased from baseline to Week 2 (+18.2 ms, *P* = 0.037), with concurrent gains in standard deviation of normal-to-normal intervals (SDNN) and rMSSD during exertion windows (+47.3 ms and +56.8 ms, both *P* = 0.037). Adding circadian alignment yielded specific nocturnal benefits: SDNN (+56.7 ms, *P* = 0.039), rMSSD (+47.6 ms, *P* = 0.0078), low-frequency (LF) power (+6236 ms², *P* = 0.039), and high-frequency (HF) power (+6812 ms², *P* = 0.0078) during sleep increased from Week 1 to Week 3. Adherence was 68% for walks, 74% for naps, and 47% for circadian timing; over half perceived better sleep quality and steadier energy levels.

**Conclusion:**

General advice improved overall autonomic regulation, whereas circadian-aligned recommendations specifically enhanced nocturnal autonomic function. These short-term physiological and perceived gains support the potential relevance of circadian biofeedback as a behavioural strategy in chronic heart failure.

## Graphical Abstract

**Graphical Abstract:**
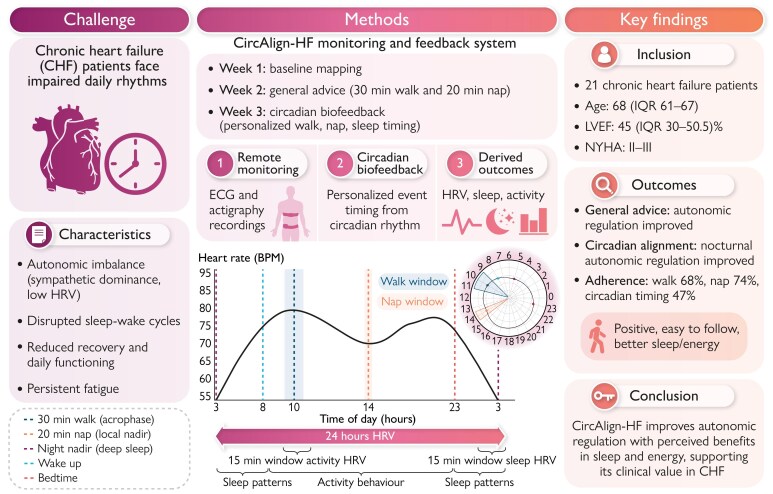

## Introduction

### Background and rationale

Chronic heart failure (CHF) is a major health concern, affecting over 64 million people worldwide and contributing to significant morbidity, mortality, and healthcare costs.^1^ Despite advances in pharmacological management, many patients experience persistent fatigue, impaired functional recovery, and reduced quality of life, often linked to autonomic imbalance and disrupted sleep–wake cycles.^2,3^ Current treatment strategies mainly target hospitalization and mortality, but they frequently overlook restoration of physiological balance and overall wellbeing. Growing evidence highlights circadian disruption as both a cause and consequence of autonomic dysfunction in CHF.^4,5^ Circadian rhythms regulate cardiovascular, metabolic, and neuroendocrine functions such as heart rate (HR), blood pressure, and autonomic tone. Disruptions may arise from external lifestyle-related factors like shift work, irregular sleep, travel, or nightly noise, as well as internal mechanisms in CHF, where rising filling pressures and reduced cardiac output activate sympathetic and Renin–angiotensin–aldosterone system pathways. This overstimulation impairs the suprachiasmatic nucleus, disrupting the internal clock and contributing to circadian misalignment, reduced nocturnal recovery, daytime fatigue, and increased risk of arrhythmia, rehospitalization, and mortality.^5–7^ Interventions aimed at restoring circadian alignment may therefore represent a novel approach to strengthen cardiovascular resilience and promote behavioural stability in CHF.

### Circadian rhythms and autonomic function in CHF

Circadian regulation of cardiovascular physiology is mediated by a hierarchical clock system, in which the suprachiasmatic nucleus synchronizes peripheral clocks in the heart, vasculature, and autonomic nervous system through neural, humoral, and behavioural cues. This system governs 24 h rhythms in HR, blood pressure, myocardial contractility, vascular tone, and inflammatory signalling, shaping daily patterns of cardiovascular risk and recovery.^4,8,9^ In CHF, these rhythms are typically dampened rather than abolished, suggesting preserved but weakened circadian control that may remain amenable to behavioural or therapeutic entrainment.^4,9,10^

Multiple mechanisms contribute to circadian misalignment in CHF. Chronic sympathetic overactivity and reduced parasympathetic tone blunt normal day–night oscillations in autonomic function and heart rate variability, while altered visceral afferent feedback from the failing heart may disrupt central clock signalling and desynchronize peripheral cardiac clocks;^4,9^ which is a strong predictor of adverse outcomes.^5,6,8,11^ At the same time, sleep-related mechanisms play a critical role. Nocturnal fluid redistribution can exacerbate sleep-disordered breathing, including obstructive and central sleep apnoea, introducing intermittent hypoxia and recurrent arousals that provoke nocturnal sympathetic surges and further destabilize circadian regulation.^4,12^

Beyond autonomic and sleep-related pathways, behavioural and environmental factors such as irregular timing of physical activity, daytime napping, meals, and light exposure may further desynchronize peripheral clocks from the central pacemaker, even in patients receiving optimal medical therapy.^4,13^ Recent state-of-the-art reviews highlight that circadian disruption in cardiovascular disease arises from the interaction of molecular clock perturbations, autonomic imbalance, sleep disturbance, and lifestyle-related timing cues.^4,9,14^ Together, these mechanisms provide a strong rationale for circadian-informed interventions that target daily behavioural timing as a complementary strategy to restore physiological rhythmicity in heart failure.

Recent advances in wearable sensing technologies enable continuous, real-world assessment of HR, HRV, physical activity, and sleep–wake patterns. When combined with analytical approaches such as cosinor modelling, these data allow characterization of individual circadian profiles and create opportunities to translate physiological rhythms into personalized, behaviourally actionable feedback.^6,15^

### Circadian biofeedback as a self-management tool in CHF

Digital health tools that deliver personalized feedback have improved disease self-management across chronic conditions.^16,17^ In CHF, however, current strategies focus mainly on symptom recognition and lifestyle guidance, such as weight monitoring, sodium restriction, and fluid control,^16^ while rarely considering the timing of behaviour or physiological rhythms. In CHF, where circadian rhythms are preserved but blunted,^10^ aligning activity, rest, and sleep with individual circadian profiles may enhance autonomic flexibility and recovery. Moderate physical activity and short, well-timed naps have both been shown to improve HRV and parasympathetic tone in healthy and athletic populations, whereas misaligned behaviours can destabilize rhythms and impair sleep quality.^18–23^ Despite this promise, circadian-aligned interventions in CHF remain largely unexplored, with prior efforts focused mainly on pharmacologic chronotherapy or laboratory studies.^4,14^ To date, no study has tested wearable-guided circadian feedback as a personalized self-management tool in this population. In this context, circadian biofeedback is defined as interpreted physiological feedback delivered at a behavioural timescale, rather than real-time closed-loop modulation of autonomic function. Unlike classical HRV biofeedback, which trains autonomic responses through moment-to-moment breathing or relaxation control, circadian biofeedback translates longitudinal physiological rhythms into guidance on when to perform daily behaviours such as activity, rest, and sleep. This approach emphasizes temporal alignment with endogenous circadian patterns rather than acute physiological regulation.

### Objective

This study evaluated the physiological effects and clinical value of a wearable-guided circadian feedback tool for patients with CHF. The primary objective was to determine whether circadian-aligned guidance on activity, rest, and sleep improves autonomic regulation across 24 h, nocturnal, and activity-related domains. Secondary objectives were patient-centred and behavioural outcomes, assessed by a structured questionnaire (study experience, adherence to specific advice, and perceived effects) and by actigraphy (sleep, activity).

## Methods

### Study population

This single-centre, crossover study investigated the effects of a wearable-guided circadian feedback intervention in adults (≥18 years) with stable CHF (NYHA class II-III) attending the cardiology outpatient clinic of the Máxima Medical Centre (Veldhoven and Eindhoven, the Netherlands). Patients were eligible regardless of heart failure aetiology or left ventricular ejection fraction. Recruitment was coordinated through treating cardiologists, after which the coordinating investigator provided verbal and written study information. Exclusion criteria included permanent atrial fibrillation (due to uncertain data quality), inability to perform daily physical activities (e.g. orthopaedic or neurological limitations), and inability to wear sensors (e.g. dermatological conditions). All participants provided written informed consent. The study protocol was reviewed by the local medical ethics committee of Máxima Medical Centre, which determined that the study did not fall under the Dutch Medical Research Involving Human Subjects Act (WMO) and issued a formal waiver indicating that prospective trial registration was not required (reference number N25.006). All procedures were conducted in accordance with the principles of the Declaration of Helsinki.

### Study instruments

#### CircAlign-HF monitoring and feedback system

**Figure 1:**
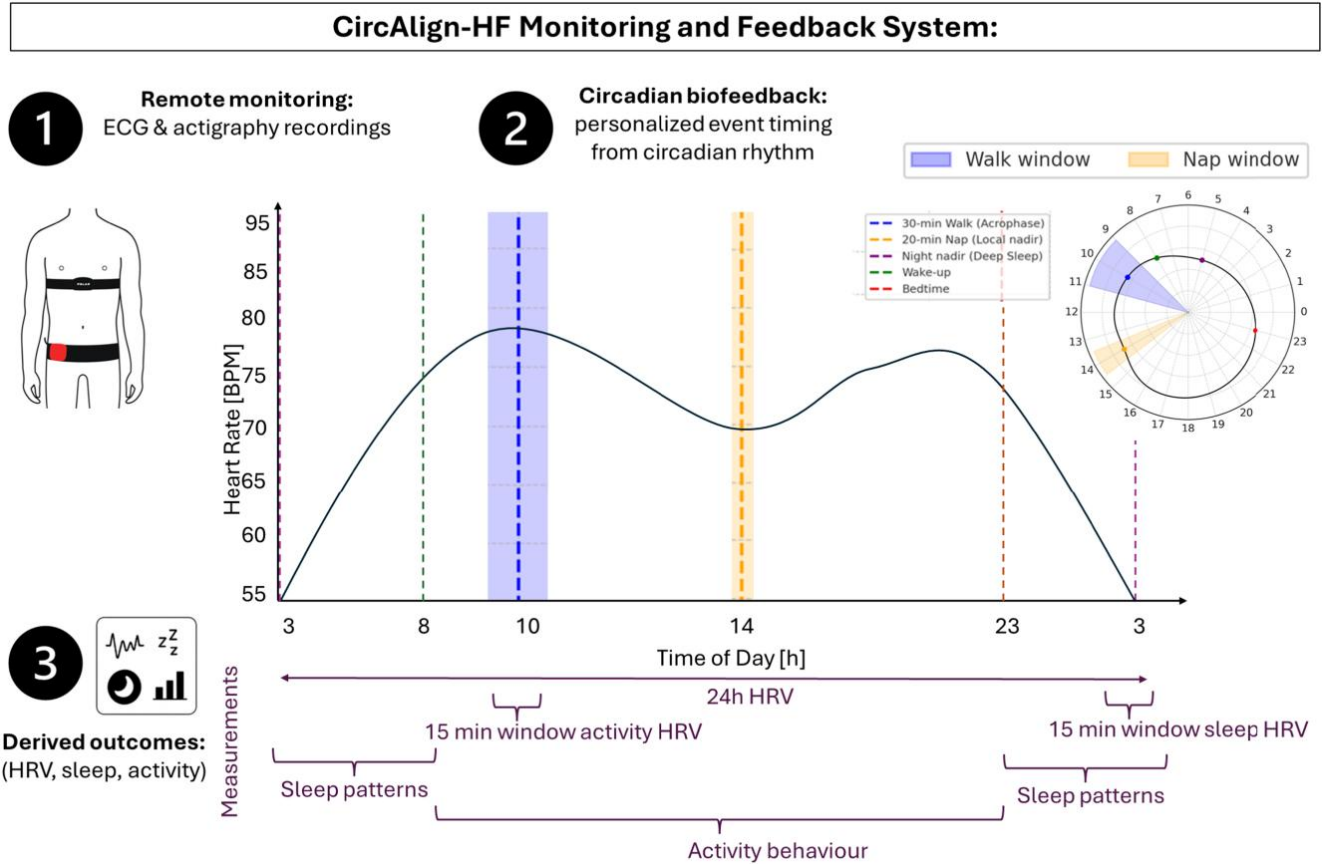
The CircAlign-HF monitoring and feedback system. The system integrates wearable devices with a feedback algorithm to support personalized circadian biofeedback in patients with chronic heart failure. (1) Remote monitoring: continuous ECG (Polar® H7) and actigraphy (ActiGraph wGT3X-BT®) recordings provide data on heart rate, HRV, activity, and sleep. (2) Circadian biofeedback: cosinor analysis of HR combined with 24 h averaged HR profiles identifies individualized acrophase (walk window), the local daytime minimum or ‘daytime dip’ (nap window), and sleep–wake slopes (bedtime/wake-up). The nocturnal nadir (global minimum) is distinguished separately as a marker of deep sleep and nocturnal recovery. (3) Derived outcomes: derived outcomes across autonomic regulation, sleep, and activity domains are translated into clinically interpretable recommendations for both individuals with CHF and clinicians to guide self-management and wellbeing.

The CircAlign-HF system integrates wearable devices with a custom analysis pipeline to provide interpretable feedback on circadian regulation, autonomic function, and sleep–wake behaviour (*Figure 1*). The pipeline comprises three components:

1. **Remote monitoring.**
  Patients continuously wore two devices (except during bathing): an ActiGraph WGT3X-BT® at the waist to assess activity and sleep–wake behaviour, and a Polar® H7 chest strap to record R–R intervals for HR-cycles and HRV analysis. Together, these devices provided continuous data on circadian rhythms, autonomic activity, sleep, and activity behaviour. Both devices have been extensively validated for their intended applications: the Polar® H7 chest strap shows high agreement with clinical-grade ECG for RR interval detection and HRV analysis in resting and ambulatory conditions,^24^ while the ActiGraph wGT3X-BT® is a widely validated research-grade accelerometer for assessing physical activity and sleep–wake patterns across healthy and clinical populations.^25,26^
2. **Circadian biofeedback.**
  Circadian biofeedback refers to the delivery of interpreted physiological feedback based on longitudinal circadian patterns, aimed at guiding the timing of daily behaviours rather than providing real-time physiological control. Accordingly, CircAlign-HF does not provide closed-loop or moment-to-moment feedback, but generates weekly, personalized timing recommendations for activity, naps, and sleep based on aggregated wearable data. This approach translates continuous physiological monitoring into actionable behavioural guidance at a circadian timescale.

To operationalize circadian biofeedback, HR data were aggregated into hourly averages and fitted with a cosinor model using gamma distribution, following the approach of Doyle *et al*.^15^ to characterize circadian parameters HR mesor (rhythm-adjusted mean HR), HR amplitude (half the difference between maximum and minimum HR within the 24 h cycle), and HR acrophase (timing of peak HR). Cosinor based modelling was used primarily to derive relative timing markers, as circadian organization is preserved albeit with dampened amplitude in CHF.^10^

To capture both stable rhythms and daily variability, cosinor fits were combined with 24 h averaged HR profiles over a weekly analysis window. Feedback was updated each week, with personalized recommendations derived from the circadian metrics of the preceding week. From the weekly composite curve, individualized time windows were identified:

- **HR acrophase:** timing of maximum HR in the 24 h cycle, used to recommend the 30 min walk.
- **Local daytime minimum (‘daytime dip’):** lowest HR outside the nocturnal sleep window, typically early afternoon, used to recommend the 20 min nap.
- **Nocturnal nadir (global minimum):** timing of the lowest HR across the 24 h cycle, used to assess nocturnal autonomic function.
- **Bedtime and wake-up:** estimated from the descending and ascending slopes of the fitted HR curve, respectively, with bedtime defined as 2 h after the descending half amplitude point to allow physiological wind-down; these markers were cross-validated with actigraphy defined sleep-wake transitions.

To ensure physiological plausibility and patient safety, predefined behavioural constraints were applied. Exercise recommendations were restricted to daytime hours (08:00–17:00 h) to avoid nocturnal sympathetic activation. Power nap recommendations were limited to the post-meridiem window (12:00–17:00 h) and identified as the local minimum in a merged curve combining the cosinor fit and the smoothed 24 h HR profile. Representative individual 24 h HR profiles with corresponding cosinor fits and derived timing markers are shown in Supplementary material online Appendix I.

**(3) Derived outcomes.**
  Physiological patterns were translated into clinically interpretable feedback across three domains:
  - **Autonomic regulation:** HRV was assessed over 24 h and within 15-min windows centred on the circadian acrophase (representing peak daytime activity) and nocturnal nadir (representing deep sleep and maximal parasympathetic dominance), as determined independently from both actigraphy- and HR-based profiles. RR intervals were extracted from the Polar® H7 chest strap via ActiLife 6 software; interbeat intervals were derived, ectopic or non-physiological beats were identified based on abrupt deviations (>15%) from neighbouring intervals and corrected using interpolation, and HRV metrics were calculated from the resulting normal-to-normal (NN) interval series.
  - **Sleep:** actigraphy-derived metrics within the individualized bedtime–wake window.
  - **Activity behaviour:** actigraphy-derived step counts and cosinor parameters describing the daily mean activity level (actigraphy mesor), the extent of diurnal variation (actigraphy amplitude), and the timing of peak activity (actigraphy acrophase).

Definitions of derived outcomes across autonomic regulation, sleep, and activity behaviour are summarized in *Table 1*.

**Table 1:** Outcome measures derived from the CircAlign-HF monitoring and feedback system

Domain	Metric	Definition	Unit	Notes
**HR circadian rhythm**	HR mesor	24 h mean HR from cosinor fit	bpm	Rhythm-adjusted baseline HR
	HR amplitude	Half the peak–nadir difference	bpm	Extent of diurnal HR variation
	HR acrophase	Timing of maximum HR	h	Used for 30-min walk timing
	HR nadir	Timing of minimum HR (nocturnal)	h	Marker of deep sleep recovery
**Autonomic regulation**	SDNN 24h	Standard deviation of NN intervals	ms	24 h HRV
	rMSSD 24h	Root mean square of successive differences	ms	24 h vagal activity
	LF 24 h, HF 24 h, LF/HF 24h	Spectral HRV indices	ms², ratio	24 h autonomic balance
	HRV at HR/actigraphy acrophase	SDNN, rMSSD, LF, HF, LF/HF in 15-min window around both HR- and actigraphy- based circadian acrophase	ms, ms², ratio	Activity-related HRV
	HRV at nocturnal HR/actigraphy nadir	SDNN, rMSSD, LF, HF, LF/HF in 15-min window around both HR- and actigraphy- based circadian nocturnal nadir	ms, ms², ratio	Deep sleep HRV
**Sleep**	Total sleep time (TST)	Actigraphy-derived sleep time	min	Sleep duration
	Sleep efficiency (SE)	TST/Time in bed	%	Overall sleep quality
	Wake after sleep onset (WASO)	Wake time after first sleep onset	min	Sleep continuity
	Sleep onset latency (SOL)	Time from bedtime to sleep onset	min	Sleep initiation
	Sleep fragmentation index (SFI)	Movement-based fragmentation	−	Sleep continuity
	Restlessness	% movement epochs in sleep window	%	Proxy for sleep disturbance
**Activity behaviour**	Steps/day	Total daily step counts	counts	Physical activity
	Actigraphy mesor	Daily average activity counts (vector magnitude)	g	Baseline activity
	Actigraphy amplitude	Deviation from mesor to peak activity	g	Strength of rhythm
	Actigraphy acrophase	Timing of peak activity	h	Active phase
	Actigraphy nadir	Timing of lowest activity	h	Rest phase

The table summarizes all outcome measures used both as study endpoints and as feedback for patients and clinicians. Metrics were grouped into three domains: (i) autonomic regulation (24 h HRV, 15-min windows at circadian acrophase and nocturnal nadir), (ii) sleep (actigraphy-derived parameters within individualized bedtime–wake windows), and (iii) activity behaviour (step counts and actigraphy-based cosinor parameters).

HRV = heart rate variability; HR = heart rate; SDNN = standard deviation of NN intervals; rMSSD = root mean square of successive differences; LF = low-frequency power; HF = high-frequency power; TST = total sleep time; SE = sleep efficiency; WASO = wake after sleep onset; SOL = sleep onset latency; SFI = sleep fragmentation index.

#### Structured end-of-study questionnaire

To assess subjective patient-centred and behavioural outcomes, a structured questionnaire was used covering four domains, each comprising specific thematic categories (see Supplementary material online Appendix II for complete questionnaire):

**1. Overall experience**
  - Positive study experience
  - Burden associated with sensor use
**2. Adherence to general advice**
  - Adherence to 30-min walk using the Borg scale
  - Barriers to walking
  - Adherence to 20-min power nap
  - Experience with time-restricted naps
**3. Adherence to circadian biofeedback**
  - Adjusted walk/nap/sleep/wake recommendation windows
  - Barriers to implementing circadian advice
  - Naturally aligned daily routines
**4. Perceived effects**
  - Improved sleep quality and depth
  - More stable energy levels
  - No noticeable changes

Open-ended responses were thematically categorized to summarize participant experiences related to study burden, adherence, and perceived effects; details of the analytic procedure are provided in Supplementary material online Appendix III.

### Study protocol

The study followed a 3-week protocol, detailed below.

**Week 0: Inclusion.** Eligible patients were enrolled during outpatient visits and provided with the monitoring devices.

**Week 1: Baseline mapping.** Baseline measures of HRV, sleep, activity, and circadian parameters were obtained. Circadian phase markers were extracted to identify each participant’s acrophase (optimal timing for a 30-min walk) and local nadir (optimal timing for a 20-min nap). Data collection was fully non-invasive and did not interfere with clinical care.

**Week 2: General advice.** Participants received standardized education on three self-management strategies: (i) a daily 30-min walk at moderate intensity and (ii) a 20-min nap. Education was delivered verbally during a home visit and supported by illustrated flyers (Appendix IV). Walking intensity was guided by the Borg Scale of Perceived Exertion (scores 4–6, ‘green zone’) to prevent undertraining or overexertion. For naps, participants were instructed to rest for exactly 20 min to maximize restorative benefits while avoiding deeper sleep stages. (iii) Basic sleep hygiene advice was also provided, focusing on reducing evening light exposure and maintaining consistent bed- and wake-times.

**Week 3: Circadian biofeedback.** Personalized recommendations were generated from each participant’s circadian profile, calculated using the mean timing of circadian rhythm parameters (acrophase, nadir, and sleep–wake transitions) across all recorded days of Week 1. Advice included (i) performing the daily walk around the individual acrophase (peak activity period), (ii) napping near the local daytime nadir (lowest HR outside the sleep window), and (iii) aligning sleep hygiene with individual bedtime and wake-up markers.

**Week 4: Sensor return and end of-study questionnaire.**

At finalization of the study patients returned the CircAlign-HF monitoring system and completed the end-of-study questionnaire.

### Statistical analysis

Baseline characteristics were summarized using descriptive statistics. Continuous variables are reported as median [interquartile range (IQR)] and categorical variables as absolute counts with corresponding percentages, as data were not normally distributed.

Physiological metrics collected at Weeks 1, 2, and 3 are presented as median (IQR). Data distributions were assessed with the Shapiro–Wilk test, which indicated non-normality across variables. Accordingly, changes over time were analysed using the nonparametric Friedman test for repeated measures.

Post hoc pairwise comparisons were performed only when the Friedman test reached statistical significance. Comparisons were conducted for the following deltas: Week 2—Week 1 (ΔWk2–Wk1), Week 3—Week 1 (ΔWk3–Wk1), and Week 3—Week 2 (ΔWk3–Wk2), using Dunn’s test with Šidák correction to adjust for multiple comparisons. Statistical significance was defined as *P* < 0.05 (Šidák-corrected).

All analyses were conducted in SPSS (version 29.0) and MATLAB (version 2024b).

#### Data completeness and analysis windows

Wearable data were considered valid when at least 20 h of continuous ECG and actigraphy recordings were available within a 24 h period. Days with insufficient wear-time (<20 h) were excluded from weekly summaries and HRV analyses. For each study week, physiological metrics were computed from the remaining valid days only. Twenty-four-hour HRV metrics were calculated using fixed midnight-to-midnight windows to ensure consistency across participants and study phases. No changes in guideline-directed heart failure medication occurred during the study period.

## Results

### Participants

A total of 21 patients with CHF were enrolled, of whom 19 (90.5%) were male. The median age was 68 years (IQR 61–76), and the median BMI was 26.9 kg/m² (IQR 24.2–29.7). Median left ventricular ejection fraction was 45% (IQR 30–50.5), distributed across heart failure with reduced ejection fraction (HFrEF, 38.1%), heart failure with mildly reduced ejection fraction (HFmrEF, 14.3%), heart failure with preserved ejection fraction (HFpEF, 14.3%), and heart failure with improved ejection fraction (HFimpEF, 33.3%). The predominant aetiologies were ischaemic cardiomyopathy (33.3%), tachycardiomyopathy (28.6%), and dilated cardiomyopathy (14.3%). Most participants were in NYHA class II (95.2%), with only one in class III.

All patients received guideline-directed medical therapy, including universal use of SGLT2 inhibitors (100%). Most were treated with β-blockers (85.7%) and mineralocorticoid receptor antagonists (85.7%), while 57.1% received sacubitril/valsartan. Common comorbidities were atrial fibrillation (71.4%) and hypertension (33.3%). Device therapy was limited to two patients (9.5%), both with an implantable cardioverter-defibrillator. Full patient characteristics are presented in *Table 2*.

**Table 2:** Baseline characteristics (*n* = 21)

**Age (years)**	68 (61–76)
**Gender**	
** Male**	19 (90.5)
** Female**	2 (9.5)
**Weight (kg)**	89 (78–96.8)
**Height (cm)**	176 (173–181)
**BMI (kg/m^2^)**	26.88 (24.22–29.74)
**LVEF (%)**	45 (30–50.5)
**HF type**	
** HFrEF**	8 (38.1)
** HFmrEF**	3 (14.3)
** HFpEF**	3 (14.3)
** HFimpEF**	7 (33.3)
**HF aetiology**	
**Ischaemic cardiomyopathy**	7 (33.3)
** Tachycardiomyopathy**	6 (28.6)
** Dilated cardiomyopathy**	3 (14.3)
** Hypertrophic cardiomyopathy**	1 (4.8)
** Diastolic dysfunction**	2 (9.5)
** Aetiology unclear**	2 (9.5)
**NYHA classification**	
** Class I**	0
** Class II**	20 (95.2)
** Class III**	1 (4.8)
** Class IV**	0
**Medication**	
** Sacubitril/valsartan**	12 (57.1)
** ACE-inhibitor**	3 (14.3)
** Angiotensin receptor blockers**	3 (14.3)
** β-blocker**	18 (85.7)
** Mineralocorticoid receptor antagonist**	18 (85.7)
** SGLTII-inhibitor**	21 (100)
** Diuretics**	9 (42.9)
** Amiodarone**	5 (23.8)
** Digoxin**	0
** Ivabradine**	1 (4.8)
** Flecainide**	0
** Verapamil**	0
** Thrombocyte aggregation inhibitor**	1 (4.8)
** Statin**	7 (33.3)
** Statin + Ezetimibe**	2 (9.5)
**Comorbidities**	
** Hypertension**	7 (33.3)
** Diabetes Mellitus**	2 (9.5)
** Hypercholesterolemia**	4 (19)
** CVA/TIA**	3 (14.3)
** PAD**	3 (14.3)
** Asthma**	2 (9.5)
** COPD**	2 (9.5)
** AF**	15 (71.4)
** DVT**	2 (9.5)
** OSA**	3 (14.3)
**Device**	
** PM**	0
** ICD**	2 (9.5)
** None**	0

BMI, body mass index; LVEF, left ventricular ejection fraction; HF, heart failure; HFrEF, heart failure with reduced ejection fraction; HFmrEF, heart failure with mildly reduced ejection fraction; HFpEF, heart failure with preserved ejection fraction; HFimpEF, heart failure with improved ejection fraction; eci.; NYHA, New York Heart Association; CVA/TIA, cerebrovascular accident/transient ischaemic attack; PAD, peripheral arterial disease; COPD, chronic obstructive pulmonary disease; AF, atrial fibrillation; DVT, deep vein thrombosis; OSA, obstructive sleep apnoea; PM, pacemaker; ICD, implantable cardioverter-defibrillator. Values are presented as number (%) or median [interquartile range (IQR)].

### Adherence to intervention components

Adherence to the general behavioural recommendations was high. Most participants integrated the daily 30 min walk (68%) and the 20 min power nap (74%) into their daily routines during the intervention period. Adherence to circadian timed recommendations was more variable; approximately 47% of participants reported adjusting the timing of walks and naps to the suggested circadian windows. Common barriers included fixed work or family schedules, weather conditions, and pre-existing routines. These adherence patterns are further detailed in the qualitative analysis (Section 3.4).

### Autonomic regulation

#### 24 h HRV

**Figure 2:**
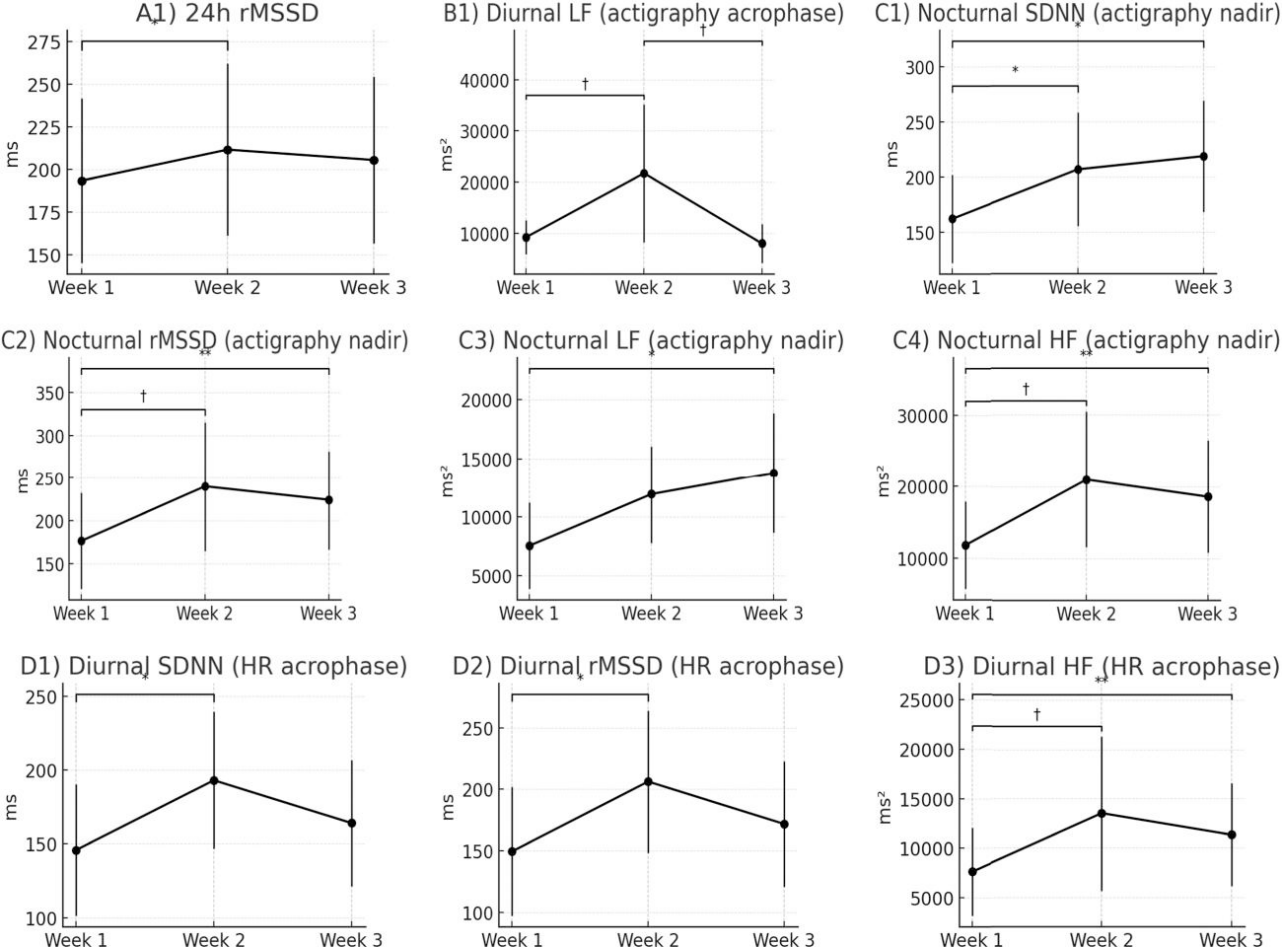
**Changes in autonomic regulation by circadian-timed HRV parameters across the intervention.** Line plots show median values (dots) with IQR (error bars) for Week 1 (baseline), Week 2 (self-scheduled general advice), and Week 3 (circadian-aligned advice) for *n* = 21. (*A*) 24 h HRV: rMSSD. (*B*) Diurnal actigraphy acrophase: LF power. (*C*) Nocturnal actigraphy nadir: SDNN, rMSSD, LF, and HF power. (*D*) Diurnal HR acrophase: SDNN, rMSSD, HF power, and LF/HF ratio. Friedman tests indicated significant overall differences for all displayed parameters (*Table 4*). Only *post hoc* Dunn−Šidák pairwise comparisons are annotated in the figure: *P < 0.05, **P < 0.01, †trend (0.05 ≤ *P* < 0.10).

Across the full 24 h recordings, rMSSD showed a significant time effect (Friedman *P* = 0.030). Post hoc testing demonstrated an increase between Week 1 and Week 2 (Δ = + 18.2 ms, *P* = 0.037), with values remaining higher but no longer significantly different in Week 3 (Δ = + 12.1 ms, *P* = 0.13) (*Table 3*, *Figure 2* A1). These changes indicate modest yet measurable improvements in parasympathetic modulation after general advice, with no further gains observed following circadian biofeedback. Other indices, including SDNN, LF, HF, and LF/HF ratio, did not change significantly (Supplementary material online Appendix V  *Table 5*).

**Table 3:** Summary of key outcome metrics across study weeks

Domain	Metric	Wk1 median (IQR)	Wk2 Median (IQR)	Wk3 median (IQR)	*P* (Friedman)	Δ Wk2–Wk1 (*P*)	Δ Wk3–Wk1 (*P*)	Δ Wk3–Wk2 (*P*)
**24 h autonomic regulation—**24 h HRV	rMSSD [ms]	193.5 (96.4)	211.7 (101.1)	205.6 (97.7)	0.030*	+18.2 (0.037)*	+12.1 (0.13)	–6.1 (0.944)
**Activity peak autonomic regulation—**HRV at activity acrophase	LF power [ms²]	9266.1 (6546.4)	21 753.8 (26 891.9)	8064.4 (7556.6)	0.034*	+12 488.0 (0.055)^a^	–1201.7 (0.641)	–13 689.0 (0.078)^a^
**Nocturnal autonomic regulation—**HRV at activity nadir	SDNN [ms]	162.3 (79.6)	207.0 (102.6)	219.0 (100.8)	0.030*	+44.7 (0.0234)*	+56.7 (0.0391)*	+12.0 (0.6406)
	rMSSD [ms]	176.5 (111.1)	239.8 (150.3)	224.2 (115.2)	0.010**	+63.3 (0.0781)^a^	+47.6 (0.0078)**	–15.7 (0.547)
	LF power [ms²]	7528.0 (7305.7)	11 905.4 (8292.8)	13 764.4 (10 240.5)	0.034*	+4377.4 (0.109)	+6236.4 (0.039)*	+1859.0 (0.742)
	HF power [ms²]	11 785.9 (12 139.9)	20 997.4 (18 920.5)	18 598.4 (15 634.7)	0.010**	+9211.5 (0.055)^a^	+6812.4 (0.0078)**	–2399.0 (0.641)
**HR peak autonomic regulation—**HRV at HR acrophase	SDNN [ms]	145.9 (89.0)	193.1 (92.8)	164.1 (85.2)	0.030*	+47.27 (0.037)*	+18.18 (0.130)	–29.09 (0.250)
	rMSSD [ms]	149.5 (104.6)	206.3 (115.9)	171.8 (101.8)	0.030*	+56.8 (0.037)*	+22.3 (0.13)	–34.5 (0.641)
	HF power [ms²]	7609.2 (8888.0)	13 493.5 (15 637.4)	11 312.8 (10 372.2)	0.034*	+5884.3 (0.072)^a^	+3703.6 (0.0078)**	–2180.7 (0.742)

Values are presented as median (IQR). Friedman tests assessed overall time effects; *post hoc* Dunn–Šidák tests compared weeks. Full results for all metrics are provided in Supplementary material online Appendix V  *Table 5*.

*P < 0.05, **P < 0.01,^a^trend (*P* < 0.10).

#### Nocturnal nadir HRV

HRV parameters measured during the nocturnal nadir of activity-based circadian oscillations showed consistent improvements. SDNN increased significantly over time (Friedman *P* = 0.030), with both Week 2 (Δ = + 44.7 ms, *P* = 0.023) and Week 3 (Δ = + 56.7 ms, *P* = 0.039) showing higher values than Week 1, while no additional difference was observed between Weeks 2 and 3 (*P* = 0.641). rMSSD also showed a robust time effect (Friedman *P* = 0.010), with a significant increase from Week 1 to Week 3 (Δ = + 47.6 ms, *P* = 0.0078), but not significant from Week 1 to Week 2 (*P* = 0.078). In the frequency domain, LF power increased significantly across weeks (Friedman *P* = 0.034), driven by a gain from Week 1 to Week 3 (Δ = + 6236 ms², *P* = 0.039), but not significant from Week 1 to Week 2 (*P* = 0.109). HF power also improved (Friedman *P* = 0.010), with a significant rise from Week 1 to Week 3 (Δ = + 6812 ms², *P* = 0.0078) but not significant from Week 1 to Week 2 (*P* = 0.055) (*Table 3*, *Figure 2* C1-C4). The LF/HF ratio did not change significantly (Friedman *P* = 0.072). Collectively, these findings show significant week-to-week modulation of nocturnal HRV, with the greatest changes observed in Week 3, indicating benefits attributable to circadian biofeedback beyond general advice alone.

#### Diurnal acrophase HRV

At the HR-defined acrophase, multiple indices improved. Friedman tests showed significant time effects for SDNN (Friedman *P* = 0.030), rMSSD (Friedman *P* = 0.030), and HF power (Friedman *P* = 0.034). Post hoc analyses revealed significant increases in SDNN (Δ = + 47.3 ms, *P* = 0.037) and rMSSD (Δ = + 56.8 ms, *P* = 0.037) from Week 1 to Week 2, which were not sustained in Week 3. HF power increased significantly between Week 1 and Week 3 (Δ = + 3704 ms², *P* = 0.0078), while the increase from Week 1 to Week 2 was not significant (*P* = 0.072). The LF/HF ratio decreased significantly between Week 1 and Week 2 (Δ = −0.27, *P* = 0.039), consistent with a relative shift towards parasympathetic dominance, but returned towards baseline thereafter. At the activity acrophase, LF power showed a significant overall time effect (Friedman *P* = 0.034), with trends towards an increase from Week 1 to Week 2 (Δ = + 12 488 ms², *P* = 0.055) and a decrease from Week 2 to Week 3 (Δ = −13 689 ms², *P* = 0.078) (*Table 3*, *Figure 2* D1-D3). Together, these results indicate that autonomic modulation during exertion improved mainly after general advice in Week 2, but the effect was not consistently maintained with circadian biofeedback in Week 3.

### Patient-centred and behavioural outcomes

#### Open-ended feedback to structured interview

The structured end-of-study questionnaire was completed by 19/21 participants. Responses were grouped into four domains and quantified using LLAMA 3.0 (*Table 4*):

**1. Overall experience**
  Most participants described the study as positive, interesting, and easy to follow (79%). They appreciated the educational component and the structure it brought to daily routines. Some reported inconvenience related to the chest strap or sensor light (26%).
  1. I found it interesting and learned a lot. I now try to keep more rest and even skip a day of cycling when needed.’ (P06)
  2. It was not burdensome; it served science and gave me awareness about rest.’ (P12)
**2. Adherence to general advice**
  The majority successfully integrated the 30-min walk (68%), often using the Borg scale to pace themselves. Some shifted to cycling or stair climbing as alternatives. Barriers included leg pain, busy schedules, and beta-blocker use. The power nap was generally well received, with most adopting it regularly (74%). Several emphasized that setting a 20-min alarm was essential.
  3. The power nap was like music to my ears. Better than collapsing on the couch.’ (P03)
  4. I now nap for 20 min instead of 40–50, and I fall asleep more easily at night.’ (P07)
**3. Adherence to circadian biofeedback**
  Adherence to circadian guidance was mixed. About half (47%) adjusted walks and naps to the suggested windows, while others found strict alignment difficult due to family or work routines. Some indicated they already followed a similar rhythm naturally.
  5. The advice time was 10 h, but I usually cycled at 11 h, depending on the weather.’ (P08)
  6. I already felt my dip and rested then. That came naturally.’ (P07)
**4. Perceived effects**
  After completing the three-week protocol, more than half (53%) reported improved sleep quality, particularly deeper sleep or easier onset when naps were shortened to 20 min and bedtimes respected. Others described more stable energy and better recovery (37%). A few noticed no change, often citing the short study duration.
  7. I could sleep deeper and fall asleep faster at night.’ (P07)
  8. Three weeks was too short to notice differences.’ (P06)

**Table 4:** Summary of patient centred outcomes (*N* = 19)

Domain	Theme	*n* (% of participants)	Representative quotes
**Overall experience**	Positive study experience, easy to follow	15 (78.9)	*‘I found it interesting and learned a lot.’ (P06)*
	Burden from sensors (chest strap, light)	5 (26.3)	*‘The strap was uncomfortable at night.’ (P11)*
**Adherence to general advice**	Integrated 30-min walk using Borg scale	13 (68.4)	*‘The green zone helped me pace the walk.’ (P04)*
	Barriers to walking (pain, schedule, medication)	6 (31.6)	*‘Walking was hard because of my leg pain.’ (P15)*
	Adopted 20-min power nap	14 (73.7)	*‘The power nap was like music to my ears.’ (P03)*
	Alarm helped limit nap duration	9 (47.4)	*‘I nap 20 min instead of 50, and sleep better at night.’ (P07)*
**Adherence to circadian biofeedback**	Adjusted walk/nap to recommended windows	9 (47.4)	*‘The advice time was 9:30 h, but I cycled at 10 or 11.’ (P08)*
	Barriers (fixed routines, weather, family duties)	8 (42.1)	*‘I couldn’t always adapt; family schedules come first.’ (P13)*
	Already aligned naturally	4 (21.1)	*‘I already felt my dip and rested then.’ (P07)*
**Perceived effects**	Improved sleep quality/depth	10 (52.6)	*‘I could sleep deeper and fall asleep faster.’ (P07)*
	More stable energy/recovery	7 (36.8)	*‘I had more energy through the day.’ (P09)*
	No noticeable changes (short study duration)	3 (15.8)	*‘Three weeks was too short to notice differences.’ (P06)*

Themes are organized by domain, with frequency counts and representative quotes.

#### Sleep and activity outcomes

Actigraphy-derived sleep parameters (total sleep time, sleep efficiency, sleep onset latency, wake after sleep onset, and fragmentation index) and activity metrics (daily step counts, mesor, acrophase, and nadir) remained stable across the three-week intervention (all *P* ≥ 0.05; Supplementary material online Appendix V  *Table 5*).

**Table 5:** Results of CircAlign-HF outcome measures across study weeks

Domain	Metric	Week 1 Median (IQR)	Week 2 Median (IQR)	Week 3 Median (IQR)	*P* (Friedman)	Δ Wk2–Wk1 (p)	Δ Wk3–Wk1 (p)	Δ Wk3–Wk2 (p)
**HR Circadian rhythm**	HR Mesor (bpm)	66.1 (18.6)	73.0 (15.7)	70.8 (15.4)	0.687	+6.87	+4.74	–2.14
	HR Amplitude (bpm)	12.1 (5.2)	13.8 (7.1)	14.4 (5.1)	0.882	+1.70	+2.35	+0.65
	HR Acrophase (h)	10.2 (3.7)	11.3 (5.1)	10.7 (3.1)	0.998	+1.18	+0.58	–0.60
	HR Nadir (h)	3.2 (1.2)	3.6 (1.3)	3.4 (1.7)	0.882	+0.40	+0.20	–0.20
**24 h autonomic regulation—24 h HRV**	SDNN 24 h (ms)	249.9 (94.6)	233.1 (87.1)	231.7 (89.5)	0.882	–16.82	–18.22	–1.40
	rMSSD 24 h (ms)	193.5 (96.4)	211.7 (101.1)	205.6 (97.7)	0.030*	+18.18 (0.037)*	+12.08 (0.130)	–6.10 (0.944)
	LF 24 h (ms²)	8335.0 (6007.0)	10 085.9 (6392.8)	9022.8 (5961.0)	0.607	+1750.8	+687.8	–1063.0
	HF 24 h (ms²)	12 006.0 (9698.2)	14 027.3 (9925.4)	13 692.4 (10 287.2)	0.093^a^	+2021.3	+1686.3	–334.9
	LF/HF 24h	0.8 (0.4)	0.8 (0.1)	0.7 (0.1)	0.998	+0.01	–0.06	–0.14
**Activity peak autonomic regulation—HRV at activity acrophase**	SDNN Act Peak (ms)	193.9 (116.0)	225.8 (119.0)	174.4 (96.7)	0.135	+31.93	–19.51	–51.44
	rMSSD Act Peak (ms)	196.1 (114.0)	230.3 (126.6)	171.8 (104.5)	0.223	+34.14	–24.33	–58.47
	LF Act Peak (ms²)	9266.1 (6546.4)	21 753.8 (26 891.9)	8064.4 (7556.6)	0.034*	+12 488.0 (0.055)^a^	–1201.7 (0.641)	–13 689.0 (0.078)
	HF Act Peak (ms²)	13 365.8 (9420.8)	19 643.7 (17 848.6)	12 793.0 (11 903.6)	0.093^a^	+6277.9	–572.8	–6850.7
	LF/HF Act Peak	0.8 (0.4)	1.0 (0.7)	0.8 (0.3)	0.998	+0.17	–0.04	–0.21
**Nocturnal autonomic regulation—HRV at activity nadir**	SDNN Act Nadir (ms)	162.3 (79.6)	207.0 (102.6)	219.0 (100.8)	0.030*	+44.71 (0.023)*	+56.73 (0.039)*	+12.02 (0.641)
	rMSSD Act Nadir (ms)	176.5 (111.1)	239.8 (150.3)	224.2 (115.2)	0.010**	+63.27 (0.078)^a^	+47.63 (0.008)**	–15.65 (0.547)
	LF Act Nadir (ms²)	7528.0 (7305.7)	11 905.4 (8292.8)	13 764.4 (10 240.5)	0.034*	+4377.4 (0.109)	+6236.4 (0.039)*	+1859.0 (0.742)
	HF Act Nadir (ms²)	11 785.9 (12 139.9)	20 997.4 (18 920.5)	18 598.4 (15 634.7)	0.010**	+9211.5 (0.055)^a^	+6812.4 (0.008)**	–2399.0 (0.641)
	LF/HF Act Nadir	1.0 (0.5)	0.8 (0.3)	0.8 (0.3)	0.072^a^	–0.17	–0.17	0.00
**HR peak autonomic regulation—HRV at HR acrophase**	SDNN HR Peak (ms)	145.9 (89.0)	193.1 (92.8)	164.1 (85.2)	0.030*	+47.27 (0.037)*	+18.18 (0.130)	–29.09 (0.250)
	rMSSD HR Peak (ms)	149.5 (104.6)	206.3 (115.9)	171.8 (101.8)	0.030*	+56.82 (0.037)*	+22.31 (0.130)	–34.50 (0.641)
	LF HR Peak (ms²)	6659.2 (8273.0)	8807.8 (8105.3)	8280.9 (7902.7)	0.093	+2148.6	+1621.7	–526.9
	HF HR Peak (ms²)	7609.2 (8888.0)	13 493.5 (15 637.4)	11 312.8 (10 372.2)	0.034*	+5884.3 (0.072)^a^	+3703.6 (0.008)**	–2180.7 (0.742)
	LF/HF HR Peak	1.1 (0.3)	0.8 (0.4)	0.9 (0.3)	0.072^a^	–0.27	–0.15	+0.13
**Nocturnal autonomic regulation—HRV at HR nadir**	SDNN HR Nadir (ms)	179.4 (121.0)	187.3 (90.4)	186.0 (88.2)	0.197	+7.93	+6.55	–1.38
	rMSSD HR Nadir (ms)	198.3 (146.0)	211.6 (126.1)	197.0 (111.3)	0.197	+13.30	–1.28	–14.58
	LF HR Nadir (ms²)	6724.4 (8519.6)	9865.1 (6458.9)	9821.3 (8334.5)	0.417	+3140.7	+3096.9	–43.8
	HF HR Nadir (ms²)	17 328.3 (18 515.2)	14 874.7 (12 317.6)	14 601.5 (13 272.2)	0.607	–2453.6	–2726.8	–273.2
	LF/HF HR Nadir	0.7 (0.2)	0.9 (0.3)	0.7 (0.1)	0.223	+0.13	–0.02	–0.15
**Sleep**	TST (min)	395.6 (29.7)	389.1 (50.9)	397.7 (35.4)	0.882	–6.51	+2.06	+8.57
	SE (%)	77.6 (5.8)	76.3 (10.0)	78.0 (6.9)	0.882	–1.28	+0.40	+1.68
	SOL (min)	20.1 (10.5)	20.1 (17.9)	23.5 (11.4)	0.135	+0.03	+3.48	+3.44
	WASO (min)	85.6 (33.7)	95.7 (41.7)	80.5 (26.2)	0.250	+10.14	–5.07	–15.21
	SFI (–)	23.8 (7.6)	22.8 (5.5)	23.0 (4.9)	0.687	–0.99	–0.76	+0.23
**Activity behaviour**	Activity Mesor (g)	0.7 (0.6)	0.7 (0.3)	0.8 (0.3)	0.993	0.00	+0.10	+0.10
	Activity Amplitude (g)	0.75 (0.4)	1.1 (0.6)	0.8 (0.4)	0.093^a^	+0.35	+0.05	–0.30
	Activity Acrophase (h)	9.6 (1.1)	9.5 (1.3)	9.7 (1.0)	0.687	–0.14	+0.11	+0.25
	Activity Nadir (h)	2.8 (2.0)	2.4 (1.3)	2.0 (1.4)	0.135	–0.40	–0.80	–0.40
	Steps/day	5266.1 (3491.6)	5779.6 (3934.6)	5955.4 (3756.0)	0.882	+513.5	+689.3	+175.9

Values are shown as median (IQR). *P*-values are from Friedman tests, with post-hoc Dunn–Šidák pairwise comparisons reported only when the Friedman test was significant (Δ = change in median).

*P < 0.05, **P < 0.01,^a^trend (*P* < 0.10).

#### Circadian phase and behaviour alignment

Cosinor analysis showed no significant changes in circadian phase across the study (all *P* ≥ 0.05; *Table 3*). HR acrophase and nadir, as well as actigraphy acrophase and nadir, remained stable. This suggests participants were already aligned with their endogenous rhythms, or that adherence to circadian-guided advice was limited, minimizing behavioural shifts in Week 3. This is in line with the patient-reported 47.4% adherence.

## Discussion

### Summary of the findings

This study evaluated the physiological and clinical value of the CircAlign-HF system, a wearable-guided circadian biofeedback tool for patients with CHF. We compared usual care to two levels of intervention: (i) general advice on daily rest, activity, and sleep hygiene and (ii) circadian biofeedback-guided recommendations aligning activity, rest, and sleep timing with individual circadian rhythms. General advice alone was sufficient to improve autonomic regulation, as reflected in increased vagal tone in 24 h HRV and transient HRV gains during exertion windows. The addition of chronotherapy principles produced a more targeted benefit, specifically enhancing nocturnal autonomic regulation through a progressive increase in parasympathetic activity at night. More than half of the participants also perceived improvements in sleep quality and daily energy distribution, reinforcing the subjective relevance of the intervention. Participants generally described the intervention as positive and easy to follow, with most integrating the daily walk and nap into their routines. Adherence to circadian-aligned recommendations was more variable, often constrained by external factors or pre-existing rhythms. Together, these findings highlight the potential clinical relevance of the CircAlign-HF system as a wearable-guided behavioural tool, combining short-term physiological modulation of autonomic regulation with perceived improvements in sleep quality and daily energy distribution.

### Potential mechanisms of our findings

The observed improvements in nocturnal HRV are most plausibly explained by enhanced parasympathetic activation and reduced sympathetic drive during sleep. Structured daytime activity combined with short, well-timed naps may have reduced evening sympathetic spillover and facilitated vagal predominance at night. This interpretation is consistent with experimental work in healthy adults, where naps timed to the circadian nadir acutely increase parasympathetic activity and cognitive performance, whereas misaligned, long naps impair sleep continuity.^21–23^ In CHF, where sympathetic overactivity and blunted vagal tone are central features, even modest nocturnal restoration of vagal activity may aid myocardial recovery, improve diastolic filling, and reduce arrhythmic vulnerability by attenuating oxidative stress.^3,27^ Anchoring bedtime to the HR downslope and wake time to the HR upslope provides a physiological scaffold for sleep–wake cycles, consistent with chronobiological principles^13,28^ In CHF, better sleep quality and preserved nocturnal vagal activity are associated with improved metabolic function and prognosis,^3,12^ highlighting the potential relevance of circadian-aligned behavioural strategies for long-term outcomes.

The transient improvements in diurnal activity-related HRV suggest that the behavioural advice itself, daily moderate-intensity walking and structured rest, was sufficient to improve autonomic flexibility, independent of circadian alignment. Exercise is known to accelerate vagal reactivation post-exercise and enhance baroreflex sensitivity, both key contributors to HRV recovery.^18,19^ The absence of sustained effects in Week 3 may indicate that circadian alignment requires longer exposure or higher adherence to yield additive benefits. Partial adherence, reported by participants facing family or work constraints, likely also contributed to the attenuation and underscores the need to interpret these findings cautiously. Thus, both biological (autonomic modulation) and behavioural (structured routines) mechanisms appear to underlie the observed improvements.

Notably, objective actigraphy derived sleep metrics remained stable despite improvements in nocturnal HRV and perceived sleep quality. This apparent dissociation is not unexpected. Actigraphy primarily captures sleep duration and movement-based continuity and is limited in its ability to assess sleep depth or autonomic state.^25^ In contrast, nocturnal HRV, particularly when assessed around the circadian nadir, reflects autonomic recovery and parasympathetic predominance associated with deeper, more restorative sleep.^29^ It is therefore plausible that parasympathetic restoration and subjective sleep quality improved without detectable changes in total sleep time or sleep efficiency. Similar dissociations between subjective sleep quality, autonomic markers, and actigraphy-derived metrics have been reported in both sleep and cardiovascular research, including in heart failure populations,^30,31^ underscoring the complementary rather than interchangeable nature of these measures.

### Results in context

Our findings contribute to the growing field of digital self-management and biofeedback in chronic disease. In CHF, most digital interventions have focused on telemonitoring of symptoms, weight, or blood pressure.^32^ Platforms such as TIM-HF2 have reduced hospitalizations through reactive, alert-based systems,^33^ but these do not actively support restoration of physiological balance. Similarly, conventional cardiac rehabilitation emphasizes exercise and counselling,^34^ which can improve HRV and functional capacity but rarely incorporate chronotherapy principles such as aligning daily behaviours with circadian rhythms.

More recently, proactive physiology-based tools have emerged. HRV biofeedback (HRVB) delivered via wearables can improve vagal tone and cardiovascular recovery by guiding resonance-frequency breathing^35^ although remote HRVB trials have reported limited compliance.^36^ These studies highlight both the promise and the practical barriers to scaling biofeedback interventions. In parallel, chronomedicine platforms such as TimeTeller integrate molecular and circadian data to individualize treatment timing,^37^ but their reliance on biological sampling limits routine home implementation and these approaches have not yet been evaluated in CHF populations. Together, these developments illustrate both the potential of physiology-guided interventions and the practical challenges of translating them into scalable, home-based care.

When contextualized against this literature, the magnitude of HRV changes observed in the present study falls within the range reported for established non-pharmacological strategies. Exercise-based interventions in CHF typically report SDNN increases of approximately 15 to 40 ms and rMSSD increases of 10 to 30 ms, with considerable variability depending on baseline autonomic impairment, intervention duration, and the analytical window used.^38,39^ HRV biofeedback interventions similarly demonstrate modest improvements in rMSSD and HF power, particularly in individuals with reduced baseline vagal tone, but these effects are often constrained by adherence in remote settings.^40^

A key methodological consideration is that our analyses combined conventional 24 h HRV with short, circadian-timed windows of approximately 15 min at the activity- and HR–defined acrophase and nadir. These physiologically anchored windows are known to yield higher absolute HRV values and larger apparent week-to-week changes than full-day averages and should therefore not be interpreted as directly equivalent to 24 h Holter-derived metrics. Rather, they provide enhanced sensitivity to state-dependent autonomic modulation, particularly during sleep, which is increasingly recognized as a critical period for cardiovascular recovery and autonomic restoration.^3^

Against this backdrop, the CircAlign-HF system is, to our knowledge, the first to integrate continuous wearable monitoring with longitudinal circadian-aligned feedback on rest, activity and sleep in the home environment for patients with CHF. Unlike telemonitoring platforms that focus on risk detection or HRVB tools that train autonomic responses via breathing exercises, CircAlign-HF actively guides patients in structuring daily routines around their biological rhythms. Our findings show that such guidance can enhance nocturnal parasympathetic activity, counteracting the sympathetic overdrive central to CHF pathophysiology, and may promote more consolidated sleep and more stable daily activity rhythms.

### Future perspectives

Our findings open several avenues for future research.

First, clinical validation; larger trials with extended interventions and longer follow-up are required to establish whether circadian-aligned feedback produces sustained autonomic benefits and translates into meaningful outcomes such as fewer hospitalizations or improved functional capacity.

Second, refinement of the intervention; Our qualitative results suggest that strict alignment is not realistic for all patients. Incorporating flexibility in recommended time windows or embedding the algorithm into adaptive digital platforms may increase feasibility and adherence. Integration with existing digital health platforms could further enable real-time feedback, gamification, and remote monitoring, thereby enhancing engagement.

Third, targeted populations; Circadian biofeedback should also be evaluated in patients with more advanced CHF or comorbid circadian disruption (e.g. sleep apnoea, atrial fibrillation, or recent hospitalization for acute decompensation), where misalignment may be more pronounced, given that the present study primarily included NYHA class II patients receiving optimal medical therapy.

Fourth, mechanistic validation; Studies combining continuous wearable monitoring with molecular circadian markers (e.g. melatonin, cortisol) are needed to disentangle behavioural influences from central clock mechanisms. Extending the algorithm to detect early circadian destabilization could also enable proactive interventions and potentially prevent hospitalization, as demonstrated in prior telemonitoring trials.^6^

Fifth, data processing and scalability; Widespread implementation of circadian biofeedback will require scalable and transparent data-processing pipelines. Automated preprocessing of wearable data (e.g. artefact detection, wear-time validation, and missing-data handling), combined with algorithmic extraction of circadian timing markers such as acrophase, nadir, and rhythm amplitude, could enable efficient and reproducible personalization of feedback in larger populations. Importantly, these computational tools are intended to support clinicians and patients by translating physiological rhythms into interpretable timing recommendations, rather than functioning as autonomous decision systems.

Finally, broader applications; The concept of wearable-guided circadian biofeedback should be seen as a platform approach with relevance beyond CHF. By targeting autonomic imbalance and promoting structured daily rhythms, similar algorithms could be adapted to other chronic conditions (e.g. hypertension, diabetes, post-myocardial infarction recovery) and to psychiatric disorders such as depression, where daily routines are often disrupted.^41^ In this way, circadian biofeedback may evolve into a versatile digital health strategy for stabilizing physiology and behaviour across diverse diseases.

### Strengths and limitations

Key strengths of this study include its novel, patient-centred design, use of validated wearable sensors, and integration of circadian analysis with clinically interpretable feedback. The crossover design, with participants serving as their own control, enhanced sensitivity to within-subject changes. In addition, the mixed-methods approach, combining objective wearable-derived metrics with qualitative feedback, provided both mechanistic and patient-centred perspectives on feasibility and acceptability.

Limitations include the small sample size and short study duration, which constrained the ability to detect changes in behavioural outcomes or clinical endpoints. Moreover, the fixed-sequence, non-randomized design limits causal separation of time effects, general behavioural advice, and circadian-aligned recommendations, and precludes definitive attribution of observed changes to circadian alignment alone. The predominantly male, NYHA class II population may not be representative of women or patients with more advanced CHF. Adherence to circadian-aligned advice was variable, with many participants citing barriers to strict timing. Finally, the absence of biochemical or central circadian markers limited mechanistic validation of the observed physiological changes. Accordingly, findings should be viewed as hypothesis generating and supportive of physiological feasibility rather than clinical efficacy.

## Supplementary Material

ztag036_Supplementary_Data

## Data Availability

The datasets generated and analysed during the current study are not publicly available due to ethical and privacy restrictions, as participants did not provide consent for open data sharing. Pseudonymized data may be made available upon reasonable request to the corresponding author.

## Lead author biography

Valerie A.A. van Es is a PhD candidate in Computational Neuroscience at the IMT school for advanced studies Lucca, in collaboration with Máxima Medical Centre and Eindhoven University of Technology. Her research focuses on non-invasive remote monitoring and management of autonomic dysregulation in cardiovascular disease, investigating circadian regulation and heart rate variability with wearables and nearables. She holds dual MSc degrees in Medical Engineering and in Architecture, Building and Planning, and works at the interface of digital health research, human physiology, and clinical application.

## Appendix

**Appendix I Representative individual 24-h HR profile with corresponding cosinor fit and derived timing markers in a participant with chronic heart failure**

**Appendix I Representative individual 24-h HR profile and cosinor based circadian timing markers in chronic heart failure.** Hourly averaged HR (blue circles, smoothed) from continuous ECG monitoring is shown together with the fitted 24 h cosinor model (orange line). The acrophase (black square) denotes the timing of peak daytime HR and was used to guide moderate physical activity. A local daytime minimum (‘daytime dip’, red triangle), identified within the post-meridiem window (12:00–17:00 h) from a merged curve combining the cosinor fit and the smoothed 24 h profile, was used to guide short restorative naps. Wake-up and bedtime markers (vertical dashed lines) were estimated from the ascending and descending half-amplitude crossings of the fitted HR curve, with bedtime defined as 2 h after the descending midpoint to allow physiological wind-down. Exercise and nap recommendations were restricted to predefined daytime windows to prevent nocturnal sympathetic activation and misaligned sleep–wake behaviour. This figure illustrates that, despite dampened rhythm amplitude in chronic heart failure, cosinor modelling yields stable and physiologically interpretable timing markers.

## Appendix II Structured end-of-study questionnaire

**1. General experience**
  1. How would you describe your overall experience participating in this study?
  2. Did you encounter any difficulties or problems while participating?
**2. Adherence to general advice**
  3. To what extent did the information about the benefits of a daily 30-min walk at moderate intensity help you integrate walking into your daily activity pattern?
  4. To what extent did the information about the benefits of a 20-min restorative power nap help you integrate power napping into your daily routine?
**3. Adherence to circadian biofeedback**
  5. To what extent did the personalized timing advice (acrophase for walking, nadir for napping) make it easier for you to integrate these activities into your daily life?
  6. To what extent was it easier for you to perform the 30-min walk during your identified peak (acrophase) hours?
  7. To what extent did you feel a natural need to rest during your identified dip (nadir) period?
**4. Perceived effects**
  8. Did you notice any improvements in your day-night rhythms as a result of participating?
    a) Was it easier to engage in physical activity?
      b) Did you experience deeper or more restorative sleep?

## Appendix III Deterministic prompt for thematic analysis of structured end-of-study questionnaire

Open-ended responses were translated into English and pseudonymized prior to analysis. The following deterministic prompt was applied using the LLaMA 3.0 model (Meta AI, version 2025; temperature = 0). The model was used exclusively for structured categorization and frequency counting within predefined domains and was not permitted to generate interpretative or inferential content beyond the provided responses.

**Prompt used for thematic analysis:**

“You are provided with translated open-ended responses from 19 participants in a qualitative health research study. Each participant answered the same set of questions.

**Tasks:**

1. Organize all responses into the following four domains:
  (i) overall study experience,
  (ii) adoption of general advice (walk, power nap),
  (iii) feasibility of circadian-aligned advice (acrophase walk, nadir nap),
  (iv) perceived effects (day–night rhythms, physical activity, sleep).
2. Identify recurring themes across participants within each domain.
3. Provide a short frequency count for each theme (e.g., ‘12/19 participants reported…’).
4. Select one or two representative anonymized quotes (short) per theme.
5. Present the results in a structured table with the following columns:
  Domain | Theme | *n* (% of participants) | Representative quotes.”

All outputs were manually reviewed by the investigators to confirm coding accuracy, resolve ambiguities, and ensure consistency with the original responses. Any discrepancies were corrected prior to reporting.

## Appendix IV Educational materials for patient self-management

**Appendix IV Educational materials for patient self-management. (A)** Borg Scale of Perceived Exertion adapted for patient education, highlighting the green zone (scores 4–6) recommended for the daily 30-min walk. **(B)** Infographic on restorative power napping, outlining four practical steps (quiet environment, lying down, setting a 20-min alarm, and closing eyes) and a safety warning to avoid naps longer than 20 min.
