# Supplementary material for: The clinical value of circadian biofeedback in chronic heart failure
Source: Eur Heart J Digit Health. 2026 Feb 25;7(3):ztag036. doi: 10.1093/ehjdh/ztag036 (PMC12993925; doi:10.1093/ehjdh/ztag036)
Supplement: ztag036_Supplementary_Data [file ztag036_supplementary_data.docx]

***11. Appendix***

**Appendix I Representative individual 24h HR profile with corresponding cosinor fit and derived timing markers in a participant with chronic heart failure**
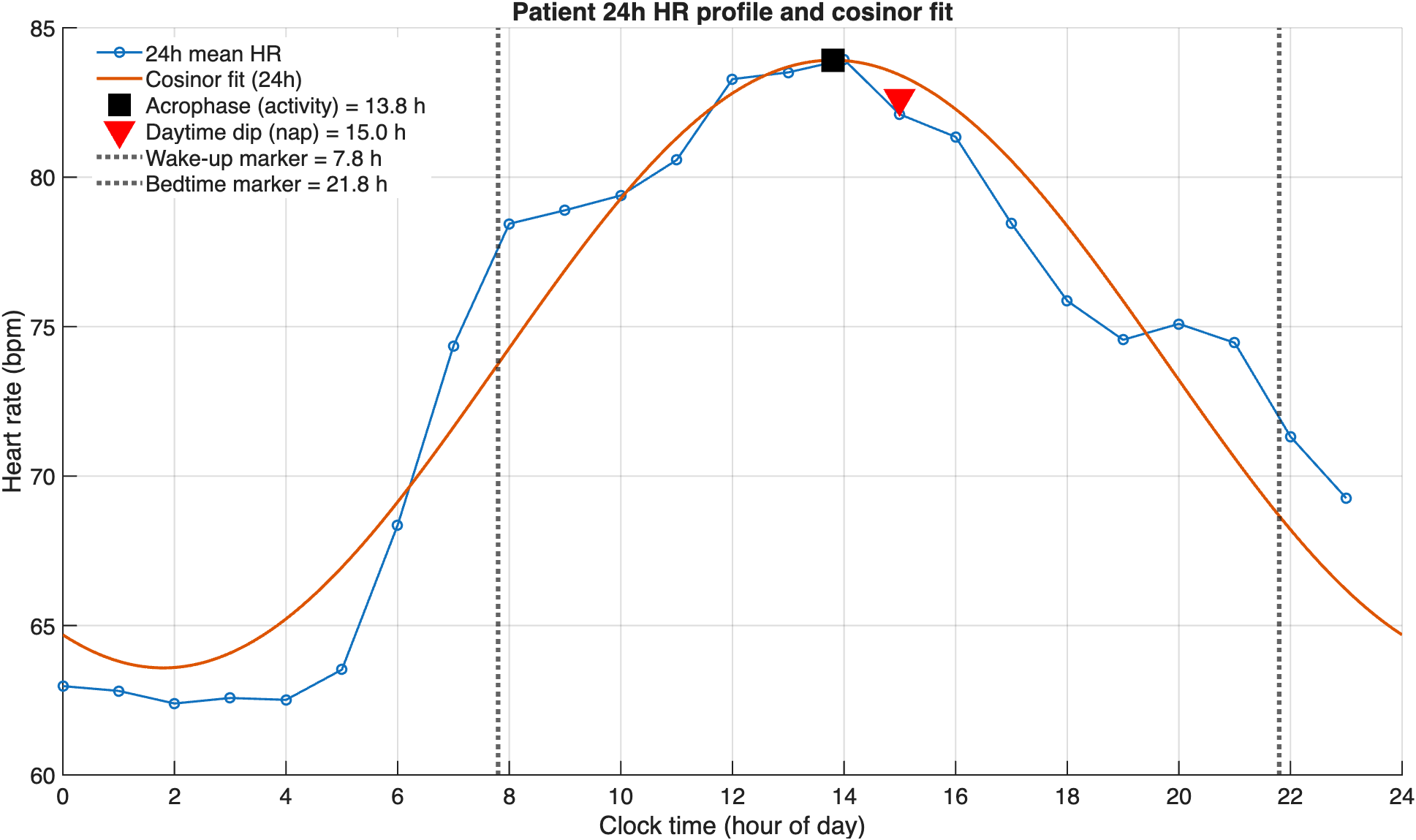


**Appendix I Representative individual 24h HR profile and cosinor based circadian timing markers in chronic heart failure.** Hourly averaged HR (blue circles, smoothed) from continuous ECG monitoring is shown together with the fitted 24h cosinor model (orange line). The acrophase (black square) denotes the timing of peak daytime HR and was used to guide moderate physical activity. A local daytime minimum (‘daytime dip,’ red triangle), identified within the post-meridiem window (12:00 - 17:00h) from a merged curve combining the cosinor fit and the smoothed 24h profile, was used to guide short restorative naps. Wake-up and bedtime markers (vertical dashed lines) were estimated from the ascending and descending half-amplitude crossings of the fitted HR curve, with bedtime defined as 2 hours after the descending midpoint to allow physiological wind-down. Exercise and nap recommendations were restricted to predefined daytime windows to prevent nocturnal sympathetic activation and misaligned sleep–wake behaviour. This figure illustrates that, despite dampened rhythm amplitude in chronic heart failure, cosinor modeling yields stable and physiologically interpretable timing markers.

**Appendix II Structured end-of-study questionnaire**

**1. General experience**

1. How would you describe your overall experience participating in this study?
2. Did you encounter any difficulties or problems while participating?

**2. Adherence to general advice**
3. To what extent did the information about the benefits of a daily 30-minute walk at moderate intensity help you integrate walking into your daily activity pattern?
4. To what extent did the information about the benefits of a 20-minute restorative power nap help you integrate power napping into your daily routine?

**3. Adherence to circadian biofeedback**
5. To what extent did the personalized timing advice (acrophase for walking, nadir for napping) make it easier for you to integrate these activities into your daily life?
6. To what extent was it easier for you to perform the 30-minute walk during your identified peak (acrophase) hours?
7. To what extent did you feel a natural need to rest during your identified dip (nadir) period?

**4. Perceived effects**
8. Did you notice any improvements in your day-night rhythms as a result of participating?
a) Was it easier to engage in physical activity?
b) Did you experience deeper or more restorative sleep?

**Appendix III Deterministic prompt for thematic analysis of structured end-of-study questionnaire**

Open-ended responses were translated into English and pseudonymized prior to analysis. The following deterministic prompt was applied using the LLaMA 3.0 model (Meta AI, version 2025; temperature = 0). The model was used exclusively for structured categorization and frequency counting within predefined domains and was not permitted to generate interpretative or inferential content beyond the provided responses.

**Prompt used for thematic analysis:**

“You are provided with translated open-ended responses from 19 participants in a qualitative health research study. Each participant answered the same set of questions.

**Tasks:**

1. Organize all responses into the following four domains:
   (i) overall study experience,
   (ii) adoption of general advice (walk, power nap),
   (iii) feasibility of circadian-aligned advice (acrophase walk, nadir nap),
   (iv) perceived effects (day–night rhythms, physical activity, sleep).
2. Identify recurring themes across participants within each domain.
3. Provide a short frequency count for each theme (e.g., ‘12/19 participants reported…’).
4. Select one or two representative anonymized quotes (short) per theme.
5. Present the results in a structured table with the following columns:
   Domain | Theme | n (% of participants) | Representative quotes.”

All outputs were manually reviewed by the investigators to confirm coding accuracy, resolve ambiguities, and ensure consistency with the original responses. Any discrepancies were corrected prior to reporting.

**Appendix IV Educational materials for patient self-management.**


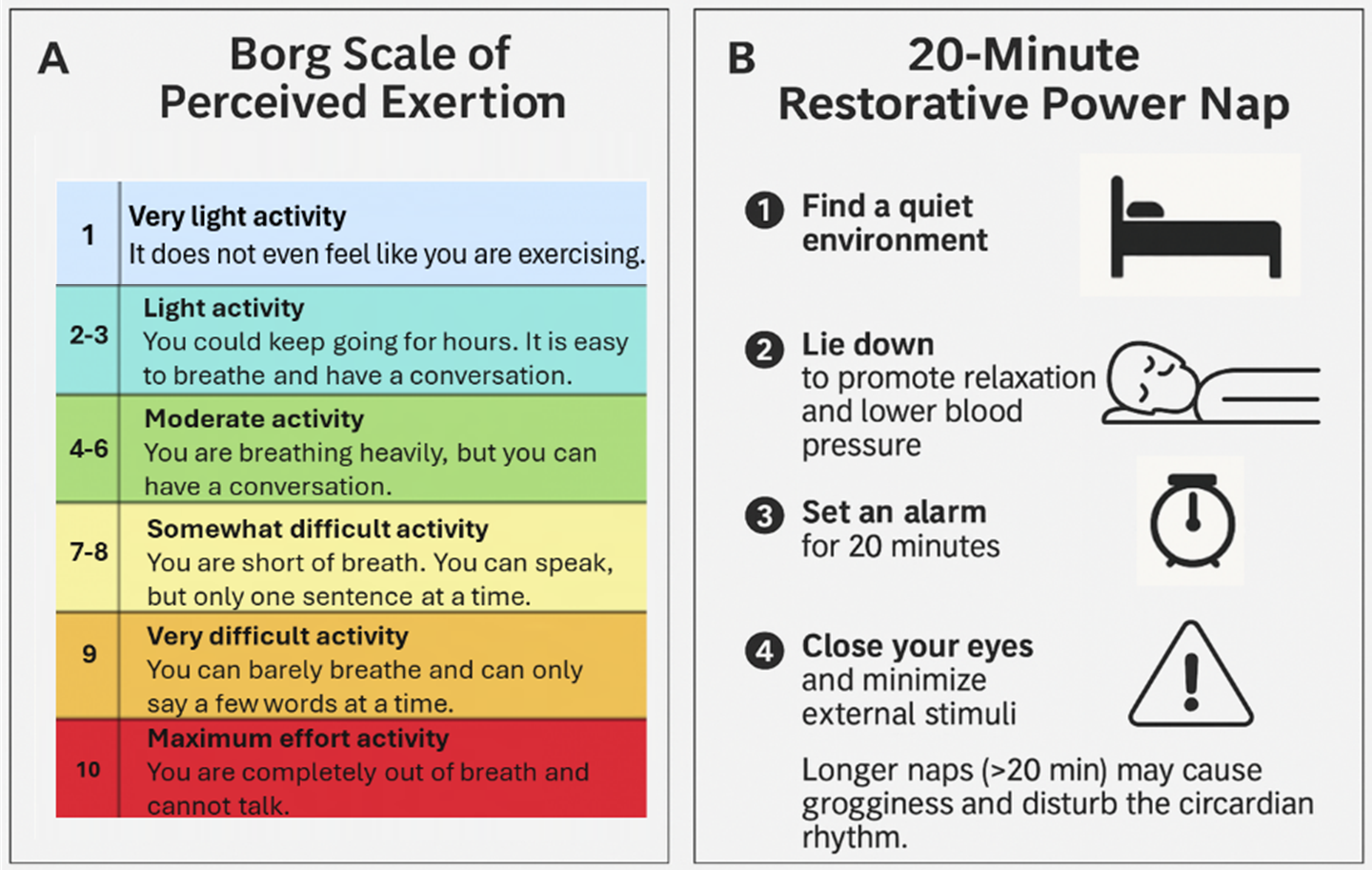


**Appendix IV Educational materials for patient self-management.** **(A)** Borg Scale of Perceived Exertion adapted for patient education, highlighting the green zone (scores 4–6) recommended for the daily 30-minute walk. **(B)** Infographic on restorative power napping, outlining four practical steps (quiet environment, lying down, setting a 20-min alarm, and closing eyes) and a safety warning to avoid naps longer than 20 minutes.

**Appendix V Summary statistics from all physiological and behavioural outcome measures across study weeks**

**Table 5. Results of CircAlign-HF outcome measures across study weeks.**

Values are shown as median (IQR). P-values are from Friedman tests, with post-hoc Dunn–Šidák pairwise comparisons reported only when the Friedman test was significant (Δ = change in median).

| **Domain** | **Metric** | **Week 1 Median (IQR)** | **Week 2 Median (IQR)** | **Week 3 Median (IQR)** | **P (Friedman)** | **Δ Wk2–Wk1 (p)** | **Δ Wk3–Wk1 (p)** | **Δ Wk3–Wk2 (p)** |
| --- | --- | --- | --- | --- | --- | --- | --- | --- |
| **HR Circadian rhythm** | HR Mesor (bpm) | 66.1 (18.6) | 73.0 (15.7) | 70.8 (15.4) | 0.687 | +6.87 | +4.74 | –2.14 |
|  | HR Amplitude (bpm) | 12.1 (5.2) | 13.8 (7.1) | 14.4 (5.1) | 0.882 | +1.70 | +2.35 | +0.65 |
|  | HR Acrophase (h) | 10.2 (3.7) | 11.3 (5.1) | 10.7 (3.1) | 0.998 | +1.18 | +0.58 | –0.60 |
|  | HR Nadir (h) | 3.2 (1.2) | 3.6 (1.3) | 3.4 (1.7) | 0.882 | +0.40 | +0.20 | –0.20 |
| **24h autonomic regulation – 24h HRV** | SDNN 24h (ms) | 249.9 (94.6) | 233.1 (87.1) | 231.7 (89.5) | 0.882 | –16.82 | –18.22 | –1.40 |
|  | rMSSD 24h (ms) | 193.5 (96.4) | 211.7 (101.1) | 205.6 (97.7) | 0.030* | +18.18 (0.037)* | +12.08 (0.130) | –6.10 (0.944) |
|  | LF 24h (ms²) | 8335.0 (6007.0) | 10085.9 (6392.8) | 9022.8 (5961.0) | 0.607 | +1750.8 | +687.8 | –1063.0 |
|  | HF 24h (ms²) | 12006.0 (9698.2) | 14027.3 (9925.4) | 13692.4 (10287.2) | 0.093† | +2021.3 | +1686.3 | –334.9 |
|  | LF/HF 24h | 0.8 (0.4) | 0.8 (0.1) | 0.7 (0.1) | 0.998 | +0.01 | –0.06 | –0.14 |
| **Activity peak autonomic regulation – HRV at activity acrophase** | SDNN Act Peak (ms) | 193.9 (116.0) | 225.8 (119.0) | 174.4 (96.7) | 0.135 | +31.93 | –19.51 | –51.44 |
|  | rMSSD Act Peak (ms) | 196.1 (114.0) | 230.3 (126.6) | 171.8 (104.5) | 0.223 | +34.14 | –24.33 | –58.47 |
|  | LF Act Peak (ms²) | 9266.1 (6546.4) | 21753.8 (26891.9) | 8064.4 (7556.6) | 0.034* | +12488.0 (0.055)† | –1201.7 (0.641) | –13689.0 (0.078) |
|  | HF Act Peak (ms²) | 13365.8 (9420.8) | 19643.7 (17848.6) | 12793.0 (11903.6) | 0.093† | +6277.9 | –572.8 | –6850.7 |
|  | LF/HF Act Peak | 0.8 (0.4) | 1.0 (0.7) | 0.8 (0.3) | 0.998 | +0.17 | –0.04 | –0.21 |
| **Nocturnal autonomic regulation – HRV at activity nadir** | SDNN Act Nadir (ms) | 162.3 (79.6) | 207.0 (102.6) | 219.0 (100.8) | 0.030* | +44.71 (0.023)* | +56.73 (0.039)* | +12.02 (0.641) |
|  | rMSSD Act Nadir (ms) | 176.5 (111.1) | 239.8 (150.3) | 224.2 (115.2) | 0.010** | +63.27 (0.078)† | +47.63 (0.008)** | –15.65 (0.547) |
|  | LF Act Nadir (ms²) | 7528.0 (7305.7) | 11905.4 (8292.8) | 13764.4 (10240.5) | 0.034* | +4377.4 (0.109) | +6236.4 (0.039)* | +1859.0 (0.742) |
|  | HF Act Nadir (ms²) | 11785.9 (12139.9) | 20997.4 (18920.5) | 18598.4 (15634.7) | 0.010** | +9211.5 (0.055)† | +6812.4 (0.008)** | –2399.0 (0.641) |
|  | LF/HF Act Nadir | 1.0 (0.5) | 0.8 (0.3) | 0.8 (0.3) | 0.072† | –0.17 | –0.17 | 0.00 |
| **HR peak autonomic regulation – HRV at HR acrophase** | SDNN HR Peak (ms) | 145.9 (89.0) | 193.1 (92.8) | 164.1 (85.2) | 0.030* | +47.27 (0.037)* | +18.18 (0.130) | –29.09 (0.250) |
|  | rMSSD HR Peak (ms) | 149.5 (104.6) | 206.3 (115.9) | 171.8 (101.8) | 0.030* | +56.82 (0.037)* | +22.31 (0.130) | –34.50 (0.641) |
|  | LF HR Peak (ms²) | 6659.2 (8273.0) | 8807.8 (8105.3) | 8280.9 (7902.7) | 0.093 | +2148.6 | +1621.7 | –526.9 |
|  | HF HR Peak (ms²) | 7609.2 (8888.0) | 13493.5 (15637.4) | 11312.8 (10372.2) | 0.034* | +5884.3 (0.072)† | +3703.6 (0.008)** | –2180.7 (0.742) |
|  | LF/HF HR Peak | 1.1 (0.3) | 0.8 (0.4) | 0.9 (0.3) | 0.072† | –0.27 | –0.15 | +0.13 |
| **Nocturnal autonomic regulation – HRV at HR nadir** | SDNN HR Nadir (ms) | 179.4 (121.0) | 187.3 (90.4) | 186.0 (88.2) | 0.197 | +7.93 | +6.55 | –1.38 |
|  | rMSSD HR Nadir (ms) | 198.3 (146.0) | 211.6 (126.1) | 197.0 (111.3) | 0.197 | +13.30 | –1.28 | –14.58 |
|  | LF HR Nadir (ms²) | 6724.4 (8519.6) | 9865.1 (6458.9) | 9821.3 (8334.5) | 0.417 | +3140.7 | +3096.9 | –43.8 |
|  | HF HR Nadir (ms²) | 17328.3 (18515.2) | 14874.7 (12317.6) | 14601.5 (13272.2) | 0.607 | –2453.6 | –2726.8 | –273.2 |
|  | LF/HF HR Nadir | 0.7 (0.2) | 0.9 (0.3) | 0.7 (0.1) | 0.223 | +0.13 | –0.02 | –0.15 |
| **Sleep** | TST (min) | 395.6 (29.7) | 389.1 (50.9) | 397.7 (35.4) | 0.882 | –6.51 | +2.06 | +8.57 |
|  | SE (%) | 77.6 (5.8) | 76.3 (10.0) | 78.0 (6.9) | 0.882 | –1.28 | +0.40 | +1.68 |
|  | SOL (min) | 20.1 (10.5) | 20.1 (17.9) | 23.5 (11.4) | 0.135 | +0.03 | +3.48 | +3.44 |
|  | WASO (min) | 85.6 (33.7) | 95.7 (41.7) | 80.5 (26.2) | 0.250 | +10.14 | –5.07 | –15.21 |
|  | SFI (–) | 23.8 (7.6) | 22.8 (5.5) | 23.0 (4.9) | 0.687 | –0.99 | –0.76 | +0.23 |
| **Activity behaviour** | Activity Mesor (g) | 0.7 (0.6) | 0.7 (0.3) | 0.8 (0.3) | 0.993 | 0.00 | +0.10 | +0.10 |
|  | Activity Amplitude (g) | 0.75 (0.4) | 1.1 (0.6) | 0.8 (0.4) | 0.093† | +0.35 | +0.05 | –0.30 |
|  | Activity Acrophase (h) | 9.6 (1.1) | 9.5 (1.3) | 9.7 (1.0) | 0.687 | –0.14 | +0.11 | +0.25 |
|  | Activity Nadir (h) | 2.8 (2.0) | 2.4 (1.3) | 2.0 (1.4) | 0.135 | –0.40 | –0.80 | –0.40 |
|  | Steps/day | 5266.1 (3491.6) | 5779.6 (3934.6) | 5955.4 (3756.0) | 0.882 | +513.5 | +689.3 | +175.9 |

*p < 0.05, **p < 0.01, †trend (p < 0.10).
